# Essentials of Surgery

**Published:** 1900-07

**Authors:** 


Essentials of Surgery. Seventh edition, with a chapter on Appendicitis. Ninety illustrations. By Edward Martin, M. D. Question Compend. Philadelphia: W. B. Saunders. 1900. [Price, $1.00 net.]

This edition contains a few corrections over the former edition and is still listed under the category of works on surgery for the hurried physician and the medical student. As a text-book for the beginner it fills a place in starting him to study surgery in a logical method, if not in a complete manner as regards to details. The pathology and diagnosis in some instances should be rewritten to meet with the more modern views. Medical students are more carefully instructed in pathology than they were ten years ago and a presupposition of a certain amount of technical knowledge would make a better book.

